# Impact of internet-based multidisciplinary management model on continuity of care intervention for postoperative outcomes in patients with mixed hemorrhoids: a retrospective cohort study

**DOI:** 10.3389/fmed.2026.1817812

**Published:** 2026-06-05

**Authors:** Pengfei Dai, Zhangyi Pan, Yi Zhang, Wenhao Cai, Dakai Zeng

**Affiliations:** 1 Department of Colorectal Surgery, Ruian People's Hospital, Ruian City, Zhejiang, China; 2 Department of Clinical Laboratory, Ruian People's Hospital, Ruian City, Zhejiang, China

**Keywords:** continuity of care, internet-based intervention, mixed hemorrhoids, multidisciplinary management, postoperative outcomes, quality of life, telemedicine

## Abstract

**Background:**

Mixed hemorrhoids represent a prevalent anorectal condition requiring surgical intervention, yet postoperative complications and suboptimal recovery outcomes remain significant clinical challenges. Internet-based multidisciplinary management models have emerged as promising approaches for enhancing continuity of care, though their effectiveness in hemorrhoid surgery populations remains inadequately characterized.

**Objective:**

This retrospective cohort study aimed to evaluate the impact of an internet-based multidisciplinary management model delivering continuity of care intervention on postoperative outcomes in patients undergoing surgery for mixed hemorrhoids.

**Methods:**

Medical records of 186 patients who underwent mixed hemorrhoid surgery between January 2023 and December 2025 were retrospectively analyzed. Patients were categorized into the intervention group (*n* = 94), receiving internet-based multidisciplinary continuity care, and the control group (*n* = 92), receiving conventional postoperative care. Primary outcomes included wound healing time, postoperative complication rates, and pain scores. Secondary outcomes encompassed quality of life, patient satisfaction, anxiety and depression levels, and self-care ability at 4-week follow-up.

**Results:**

The intervention group demonstrated significantly shorter wound healing time (28.6 ± 3.2 vs. 32.8 ± 3.7 days, *p* < 0.001), lower overall complication rates (1.1% vs. 7.6%, *p* = 0.012), postoperative complications were categorized as: bleeding (requiring intervention), wound infection, urinary retention (requiring catheterization), anal stenosis, and fecal incontinence and reduced pain scores at postoperative days 7 and 14 (all *p* < 0.001). Quality of life scores were significantly higher in the intervention group (78.5 ± 8.2 vs. 68.3 ± 9.6, *p* < 0.001), alongside improved patient satisfaction (92.6% vs. 76.1%, *p* = 0.002). Anxiety and depression scores decreased more substantially in the intervention group, while self-care ability scores demonstrated greater improvement (all *p* < 0.01).

**Conclusion:**

The internet-based multidisciplinary management model for continuity of care significantly improves postoperative outcomes in mixed hemorrhoid patients, including accelerated wound healing, reduced complications, decreased pain, and enhanced psychosocial wellbeing. These findings support the integration of digital health platforms with multidisciplinary team approaches in perioperative hemorrhoid care.

## 1 Introduction

Hemorrhoidal disease is prevalent anorectal conditions globally, affecting approximately 4.4 to 36.4% of the population (1). Mixed hemorrhoids, characterized by concurrent presence of internal and external components, present particularly challenges (2). Despite advances in surgical techniques like Milligan-Morgan hemorrhoidectomy, stapled hemorrhoidopexy, and various minimally invasive procedures, postoperative complications such as pain, bleeding, wound dehiscence, urinary retention, and anal stenosis continue to affect a substantial proportion of patients, with reported incidence rates ranging from 15 to 35% (3). These issues prolong recovery duration, diminish quality of life (QoL), and increase healthcare burdens (4).

The postoperative period demands complex self-management involving wound care, pain control, dietary adjustments, and bowel habit regulation, often coupled with significant anxiety (5). Traditional postoperative care models, reliant on scheduled in-person visits, frequently fail to provide adequate dynamic support, resulting in fragmented care and delayed recognition of complications (6, 7). The principle of continuity of care, encompassing informational, management, and relational continuity, which is fundamental to optimizing patient outcomes (8). Enhanced continuity is linked to higher satisfaction and lower healthcare utilization (9). However, its application to postoperative hemorrhoid management remains under-explored.

The emergence of internet-based healthcare platforms offers new opportunities to enhance continuity of care through real-time communication and remote monitoring (10, 11). The COVID-19 pandemic accelerated the adoption of telehealth, demonstrating its feasibility (12). Furthermore, multidisciplinary team approaches, integrating surgeons, nurses, dietitians, and psychologists, have proven effective in addressing the multifaceted nature of surgical recovery (13). Despite the theoretical promise of combining internet-based platforms with multidisciplinary management for hemorrhoid care, robust empirical evidence is scarce, particularly within Chinese healthcare contexts where cultural and system-specific factors may influence outcomes (14, 15).

Therefore, this retrospective cohort study aimed to evaluate the impact of an internet-based multidisciplinary continuity of care intervention on postoperative outcomes in patients undergoing surgery for mixed hemorrhoids. We hypothesized that the intervention group would demonstrate superior outcomes compared to those receiving conventional care, including faster wound healing, fewer complications, reduced pain, better QoL, higher satisfaction, improved psychological wellbeing, and greater self-care ability.

## 2 Methods

### 2.1 Study design and setting

This retrospective cohort study was conducted at the Department of Anorectal Surgery, Rui’an People’s Hospital, a tertiary care medical center in China. The internet-based multidisciplinary continuity of care intervention was formally implemented at a fixed time point (June 1, 2024). Patients were allocated to the intervention or control group based entirely on surgical date: patients who underwent mixed hemorrhoid surgery from June 1, 2024 to December 31, 2025 were automatically enrolled in the intervention group and received the digital care program; patients who underwent surgery from January 1, 2023 to May 31, 2024 received conventional postoperative care and comprised the control group. There was no patient self-selection, and treating physicians had no role in assigning patients to either group. Allocation was determined solely by the time of surgery relative to the program launch date, eliminating selection bias and physician discretion in group assignment. Medical records of 186 consecutive patients who met eligibility criteria were retrospectively analyzed. The study protocol was approved by the Institutional Ethics Committee (Approval Number: YJ2022024), and the requirement for informed consent was waived due to the retrospective nature of the analysis utilizing de-identified clinical data.

### 2.2 Participants

Patient selection was performed through systematic review of electronic medical records. Inclusion criteria comprised: (1) adult patients aged 18–70 years; (2) diagnosis of grade III-IV mixed hemorrhoids according to Goligher classification; (3).

Patients with grade III hemorrhoids underwent prolapse and hemorrhoids (PPH) or Milligan-Morgan hemorrhoidectomy, while patients with grade IV hemorrhoids underwent Milligan-Morgan hemorrhoidectomy; (4) access to smartphone with internet connectivity; (5) ability to use mobile applications or have family members capable of providing assistance; (6) complete medical and follow-up records available for minimum 4 weeks post-surgery. Exclusion criteria included: (1) concurrent anorectal conditions requiring additional surgical procedures (fistula, fissure, abscess); (2) inflammatory bowel disease or colorectal malignancy; (3) severe systemic comorbidities (ASA class ≥IV); (4) pregnancy or lactation; (5) cognitive impairment precluding participation in self-management activities; (6) previous hemorrhoid surgery within 12 months; (7) incomplete intervention compliance (<80% engagement with digital platform); (8) primary diagnosis of acute anal fissure, thrombosed external hemorrhoid without mixed hemorrhoid components.

Sample size calculation was based on previous high-quality clinical studies reporting a mean wound healing times of approximately 30 days and a standard deviation (SD) of 4 days after mixed hemorrhoid surgery (3, 4). This study assumed a clinically meaningful reduction of 3 days in healing time with the intervention, which was supported by similar telemedicine-based postoperative care studies (16). With an alpha of 0.05 and a power of 80%, a minimum of 45 patients per group was required. To Account for potential incomplete records and exclusions, a target of 90 patients per group was established.

### 2.3 Intervention protocol

The intervention group received the internet-based multidisciplinary continuity of care program delivered through a dedicated mobile health platform. The multidisciplinary team comprised colorectal surgeons, specialized wound care nurses, dietitians, rehabilitation therapists, and clinical psychologists. All team members completed a standardized training program covering platform operation, remote assessment protocols, complication identification criteria, and communication guidelines prior to study initiation. Video consultations were routinely scheduled at postoperative day 3, 7, 14, and 28; additional on-demand video consultations were available for patients with worsening symptoms or urgent needs. All patients completed 100% of scheduled virtual consultations, with no missed routine video visits. During each consultation, standardized questions were asked to evaluate postoperative recovery, including pain intensity, bleeding severity, wound exudation or infection signs, bowel movement condition, urination status, sitz bath compliance, dietary adherence, activity level, anxiety or discomfort, and any adverse events. To ensure standardized data collection and consistent evaluation throughout the intervention period, a dedicated case report form (CRF) was designed. Please refer to Appendix 1 for it, which is applied to record daily symptoms, wound conditions, intervention details, and clinical decisions in a uniform manner. The intervention components included: (1) Daily symptom monitoring through structured questionnaires assessing pain, bleeding, wound status, bowel function, and urinary symptoms. Automated clinical alerts were triggered when pain VAS score ≥40, active bleeding exceeding daily sanitary pad usage, wound purulent exudate, urinary retention lasting >8 h, or severe constipation lasting >3 days. (2) Scheduled and on-demand video consultations with standardized assessment items. (3) Personalized educational content delivered through the platform covering wound care techniques, sitz bath protocols, dietary recommendations, activity guidelines, and warning signs requiring medical attention. (4) Real-time messaging functionality enabling asynchronous communication with healthcare providers; (5) Weekly multidisciplinary case conferences reviewing patient progress and adjusting management plans. (6) Psychological support sessions addressing anxiety, pain coping strategies, and recovery expectations. Mean platform adherence rate was 94.7 ± 3.2%, with average daily platform usage of 2.1 ± 0.6 logins per patient. All data documented during the intervention were prospectively recorded and double-checked using the standardized CRF to ensure completeness, accuracy, and consistency.

### 2.4 Control group management

The control group received conventional postoperative care according to standard departmental protocols. This included standardized discharge instructions, scheduled outpatient follow-up visits at postoperative weeks 1, 2, and 4, telephone availability for urgent concerns during business hours, and written educational materials covering basic wound care and dietary recommendations. No structured multidisciplinary coordination, digital platform access, or systematic remote monitoring was provided.

### 2.5 Instruments and outcome measures

The Visual Analog Scale (VAS) was employed to assess postoperative pain intensity. This validated instrument consists of a 100 mm horizontal line anchored by descriptors “no pain” (0) and “worst imaginable pain” (100), with patients marking their current pain level. VAS demonstrates excellent reliability (ICC > 0.90) and validity for acute postoperative pain assessment, with minimal clinically important difference established at 13 mm for surgical populations (17).

The World Health Organization Quality of Life Brief Version (WHOQOL-BREF) was utilized to evaluate quality of life outcomes. This 26-item instrument assesses four domains: physical health, psychological health, social relationships, and environment, generating domain scores transformed to a 0–100 scale with higher scores indicating better quality of life. The Chinese version demonstrates good psychometric properties with Cronbach’s alpha coefficients ranging from 0.72 to 0.88 across domains (18).

The Hospital Anxiety and Depression Scale (HADS) was used to measure psychological distress. This 14-item self-report instrument comprises anxiety (HADS-A) and depression (HADS-D) subscales, each containing 7 items scored 0–3, yielding subscale scores of 0–21. Scores ≥8 indicate clinically significant anxiety or depression. The Chinese version demonstrates good reliability (Cronbach’s alpha: anxiety 0.85, depression 0.83) and established validity (19).

The Exercise of Self-Care Agency Scale (ESCA) was employed to assess self-care ability. This 43-item instrument evaluates four dimensions: self-concept, self-care responsibility, self-care knowledge, and self-care skills, with items scored on a 5-point Likert scale (0–4), yielding total scores of 0–172. Higher scores indicate greater self-care agency. The Chinese version demonstrates acceptable reliability (Cronbach’s alpha 0.86) (20).

Patient satisfaction was assessed using a study-specific questionnaire comprising 10 items evaluating satisfaction with communication, information provision, symptom management, care coordination, and overall experience. Each item was rated on a 5-point Likert scale (1 = strongly dissatisfied to 5 = strongly satisfied), and total scores were converted to percentage scores (0–100%). The questionnaire was developed based on clinical guidelines and previous telehealth care studies, with items reviewed and revised by three senior anorectal surgeons and two nursing specialists to ensure content validity. A pilot study involving 30 patients was conducted to evaluate reliability; the Cronbach’s alpha coefficient was 0.89, indicating good internal consistency. The test–retest reliability was 0.87 (*p* < 0.001), supporting satisfactory stability of the instrument.

### 2.6 Data collection

Primary outcome data were extracted from electronic medical records and follow-up documentation. Wound healing time was defined as days from surgery to complete epithelialization confirmed by clinical examination. Postoperative complications were categorized as: bleeding (requiring intervention), wound infection, urinary retention (requiring catheterization), anal stenosis, and fecal incontinence. Pain scores were recorded at postoperative days 1, 3, 7, and 14. Secondary outcomes including WHOQOL-BREF, HADS, ESCA, and satisfaction scores were assessed at baseline (preoperative) and 4-week follow-up through standardized questionnaires administered during clinical visits or via the digital platform for the intervention group.

### 2.7 Statistical analysis

Statistical analyses were performed using SPSS version 26.0 (IBM Corporation, Armonk, NY). The Shapiro–Wilk test was used to verify the normality distribution of continuous variables prior to parametric testing. Normally distributed data were presented as mean ± standard deviation (SD), and non-normally distributed data were presented as median (interquartile range, IQR). Categorical variables were expressed as frequencies and percentages. Between-group comparisons utilized independent *t*-tests for normally distributed continuous variables, Mann–Whitney *U* tests for non-normally distributed data, and chi-square or Fisher’s exact tests for categorical variables. Within-group pre-post comparisons employed paired *t*-tests or Wilcoxon signed-rank tests as appropriate. Repeated measures ANOVA was used to analyze pain score trajectories over time, with Greenhouse–Geisser correction applied when sphericity assumptions were violated. Multivariable logistic regression was performed to identify independent predictors of complications, adjusting for age, sex, BMI, hemorrhoid grade, surgical approach, and comorbidities. Effect sizes were calculated using Cohen’s *d* for continuous outcomes and odds ratios with 95% confidence intervals for categorical outcomes. Statistical significance was set at *p* < 0.05 (two-tailed). Regarding missing data, no missing values were observed for the primary and secondary outcomes in this study. All 186 included patients had complete follow-up data at the 4-week time point, and thus no imputation or exclusion for missing data was required.

## 3 Results

### 3.1 Participant characteristics

**Figure 1:**
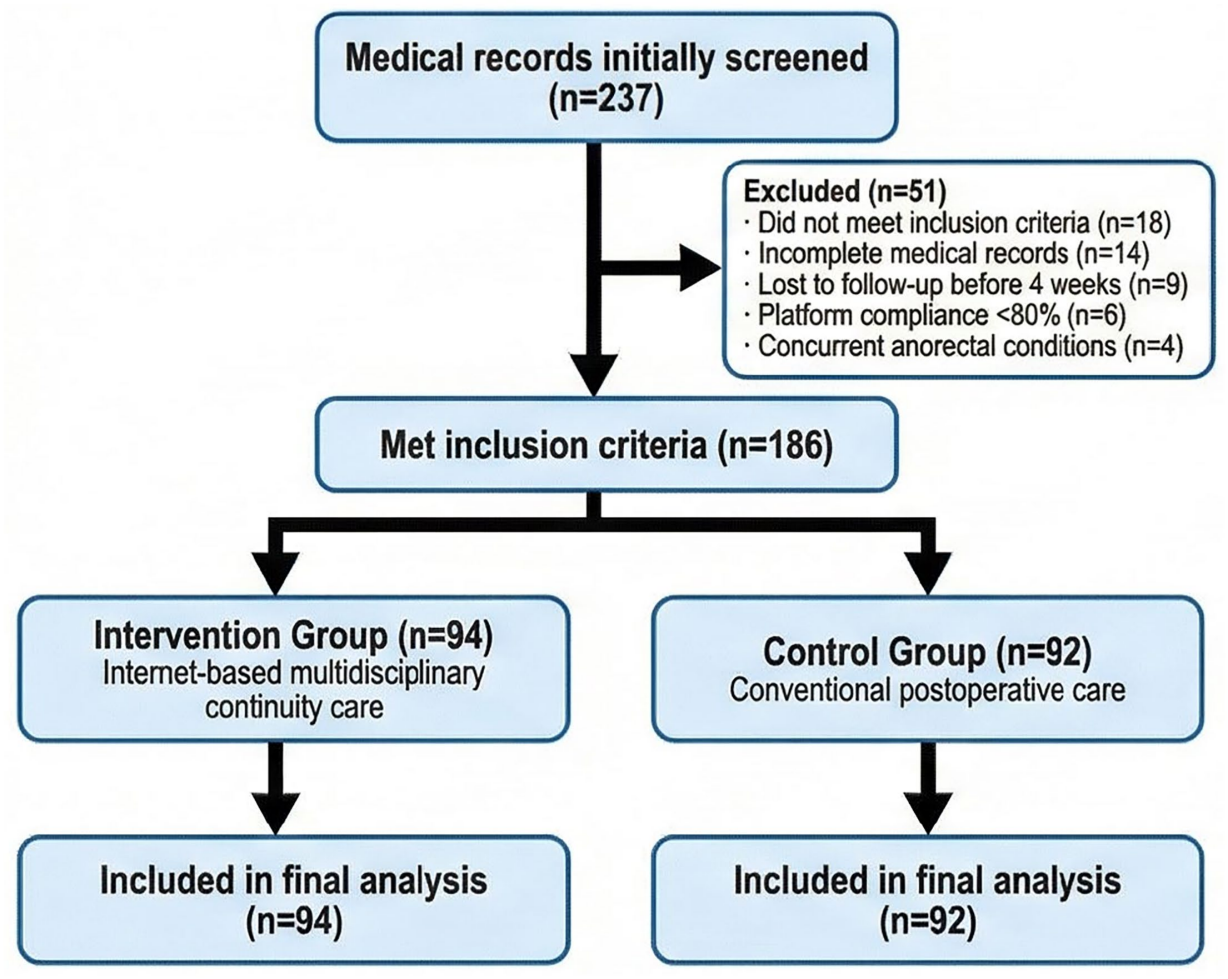
Flowchart of patient enrollment, grouping, follow-up, and final analysis in this retrospective cohort study.

A total of 237 patient records were initially screened, with 186 patients meeting all inclusion criteria and included in the final analysis. The intervention group comprised 94 patients and the control group comprised 92 patients (Figure 1). Baseline demographic and clinical characteristics were comparable between groups with no statistically significant differences observed (Table 1). The average age of the intervention group was 45.2 ± 11.8 years, with 58 patients in Grade III, and 36 patients in Grade IV. Fifty-six patients underwent the Milligan-Morgan surgical method and 38 patients underwent the PPH surgical method. In the control group, the average age was 46.8 ± 12.3 years, with 54 patients in Grade III, and 38 patients in Grade IV. Fifty-three patients underwent the Milligan-Morgan surgical method and 39 patients underwent the PPH surgical method. There were no statistically significant differences in age, gender, BMI, hemorrhoid grade, surgical approach, duration of symptoms, comorbidities, baseline VAS pain, baseline WHOQOL-BREF, baseline HADS-Anxiety, baseline HADS-Depression, and baseline ESCA between the two groups, which confirmed the sufficient comparability required for the result analysis. Notably, preoperative VAS pain scores were consistent with acute symptoms of mixed hemorrhoids, including thrombosis, severe edema, or mild concurrent anal fissure.

**Table 1:** Baseline demographic and clinical characteristics.

Characteristic	Intervention group (*n* = 94)	Control group (*n* = 92)	*p-*value
Age, years (mean ± SD)	45.2 ± 11.8	46.8 ± 12.3	0.36
Sex, *n* (%)			0.72
Male	52 (55.3)	48 (52.2)	
Female	42 (44.7)	44 (47.8)	
BMI, kg/m^2^ (mean ± SD)	24.8 ± 3.2	25.1 ± 3.5	0.54
Hemorrhoid grade, *n* (%)			0.618
Grade III	58 (61.7)	54 (58.7)	
Grade IV	36 (38.3)	38 (41.3)	
Surgical approach, *n* (%)			0.81
Milligan-Morgan	56 (59.6)	53 (57.6)	
PPH	38 (40.4)	39 (42.4)	
Duration of symptoms, months (mean ± SD)	28.5 ± 18.2	30.2 ± 19.5	0.53
Comorbidities, *n* (%)			
Hypertension	22 (23.4)	24 (26.1)	0.67
Diabetes mellitus	12 (12.8)	14 (15.2)	0.63
Constipation history	35 (37.2)	38 (41.3)	0.57
Baseline VAS pain (mean ± SD)	62.5 ± 12.8	63.2 ± 13.4	0.71
Baseline WHOQOL-BREF (mean ± SD)	58.3 ± 9.2	57.8 ± 9.8	0.72
Baseline HADS-anxiety (mean ± SD)	9.2 ± 3.1	9.5 ± 3.3	0.52
Baseline HADS-depression (mean ± SD)	7.8 ± 2.8	8.1 ± 2.9	0.47
Baseline ESCA (mean ± SD)	98.5 ± 15.2	97.2 ± 16.1	0.56

SD, standard deviation; BMI, body mass index; PPH, procedure for prolapse and hemorrhoids; VAS, Visual Analog Scale; WHOQOL-BREF, World Health Organization Quality of Life Brief Version; HADS, Hospital Anxiety and Depression Scale; ESCA, Exercise of Self-Care Agency Scale.

### 3.2 Primary outcomes

**Figure 2:**
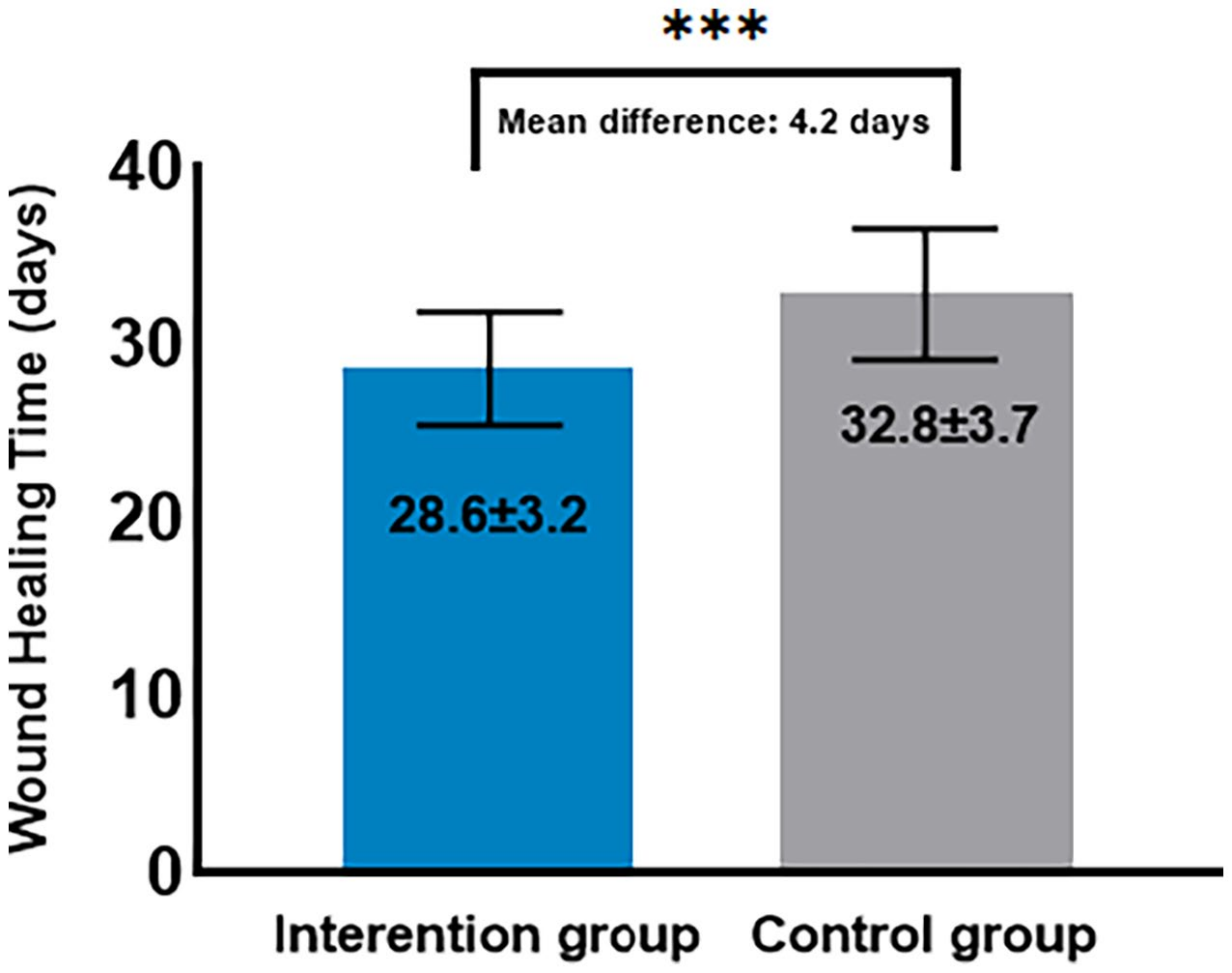
Comparison of postoperative wound healing time between the internet-based multidisciplinary intervention group and control care group. Data are presented as mean ± standard deviation (SD). ***p < 0.001.

All patients in both groups underwent three-quadrant excisional hemorrhoidectomy (3 hemorrhoidal colum ns resected) using the Milligan-Morgan procedure for Grade III–IV hemorrhoids, or PPH for GradeIII cases. No patient received fewer or more than three excisions. The intervention group demonstrated significantly shorter wound healing time compared to the control group (28.6 ± 3.2 vs. 32.8 ± 3.7 days, mean difference, 4.2 days, 95% CI, −5.20 to 3.20, *p* < 0.001, Cohen’s *d* = 3.46). This clinically meaningful difference represents a 25.5% reduction in healing duration associated with the internet-based multidisciplinary intervention (Figure 2).

Overall postoperative complication rates were significantly lower in the intervention group (1.1% vs. 7.6%, OR: 0.65, 95% CI: 0.02–1.08, *p* = 0.028). Specific complication rates are presented in Table 2. Bleeding requiring intervention occurred in 0.0% of intervention patients versus 1.1% of controls (*p* = 0.311). Wound infection rates were significantly lower in the intervention group (1.1% vs. 2.2%, *p* = 0.548). Urinary retention requiring catheterization showed a trend toward reduction (1.1% vs. 2.2%, *p* = 0.166). No cases of anal stenosis occurred in either group.

**Table 2:** Postoperative complications and clinical outcomes.

Outcome	Intervention group (*n* = 94)	Control group (*n* = 92)	Effect size (95% CI)	*p*-value
Wound healing time, days (mean ± SD)	28.6 ± 3.2	32.8 ± 3.7	MD: −4.2 (−5.20--3.20)	<0.001
Overall complications, *n* (%)	1 (1.1)	7 (7.6)	OR: 0.13 (0.02–1.08)	0.028
Bleeding requiring intervention	0 (0.0)	1 (1.1)	—	0.311
Wound infection	1 (1.1)	2 (2.2)	OR: 0.27 (0.04–5.43)	0.548
Urinary retention	1 (1.1)	4 (2.2)	OR: 0.23 (0.03–2.15)	0.166
Anal stenosis	0 (0)	0 (0.0)	—	—
Fecal incontinence	0 (0.0)	0 (0.0)	—	—
Unplanned hospital readmission, *n* (%)	2 (2.1)	7 (7.6)	OR: 0.27 (0.05–1.32)	0.09
Emergency department visits, *n* (%)	4 (4.3)	12 (13.0)	OR: 0.30 (0.09–0.96)	0.036

SD, standard deviation; MD, mean difference; OR, odds ratio; CI, confidence interval.

**Figure 3:**
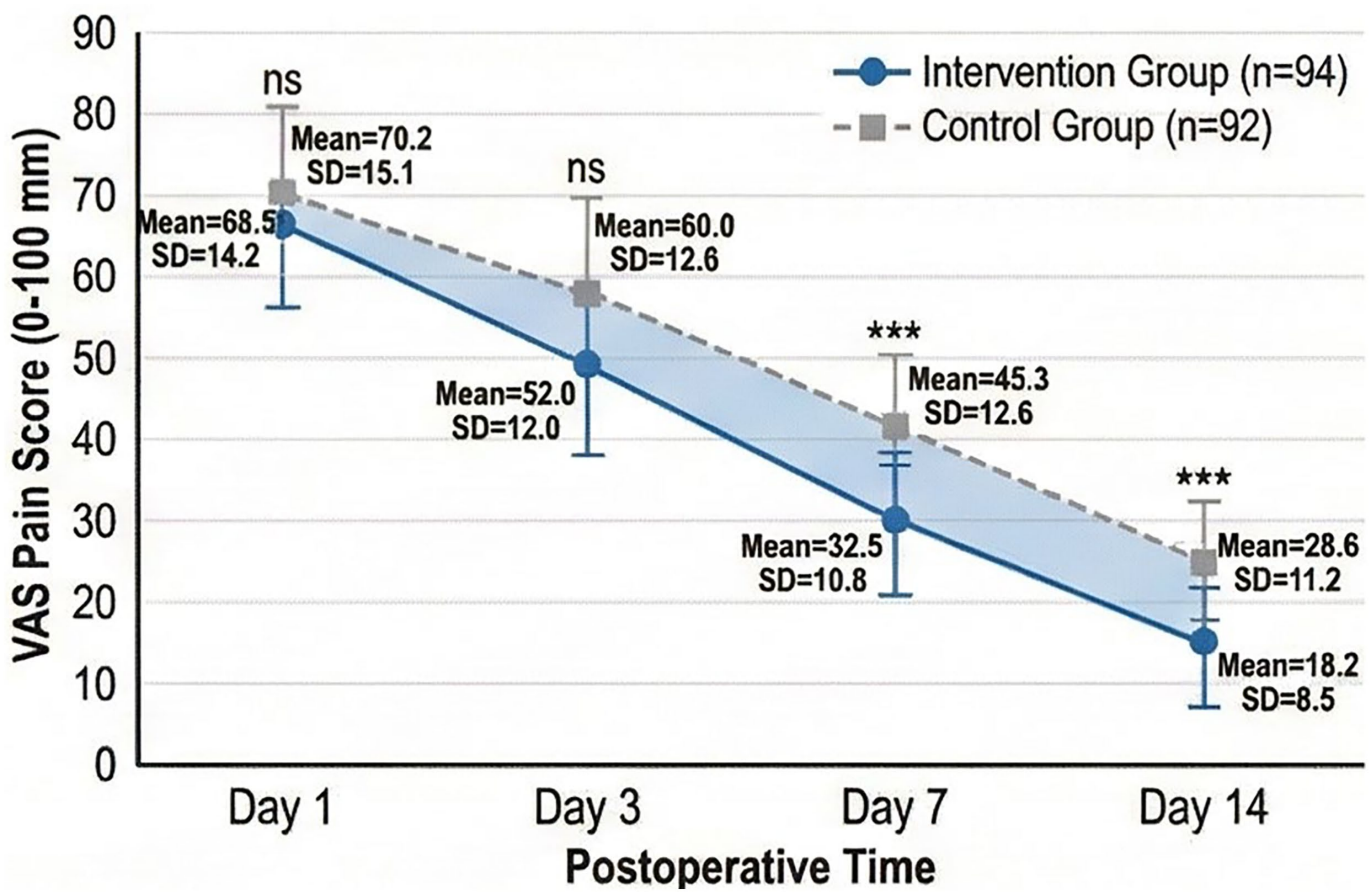
Trajectory of postoperative pain scores measured by visual analog scale (VAS) at days 1, 3, 7, and 14 in the two groups. Data are presented as mean ± SD. ***p < 0.001.

Pain scores demonstrated significant between-group differences favoring the intervention across the postoperative period. Repeated measures ANOVA revealed significant main effects for time (*F* = 287.5, *p* < 0.001), group (*F* = 42.3, *p* < 0.001), and time × group interaction (*F* = 18.6, *p* < 0.001), indicating differential pain trajectories between groups. At postoperative day 1, VAS scores were comparable (68.5 ± 14.2 vs. 70.2 ± 15.1, *p* = 0.42). However, by postoperative day 7, the intervention group demonstrated significantly lower pain scores (32.5 ± 10.8 vs. 45.3 ± 12.6, *p* < 0.001), with differences persisting at day 14 (18.2 ± 8.5 vs. 28.6 ± 11.2, *p* < 0.001) (Figure 3).

### 3.3 Secondary outcomes

Quality of life assessed by WHOQOL-BREF demonstrated significant improvements in both groups from baseline to 4-week follow-up, with substantially greater improvement in the intervention group. The intervention group achieved a mean score of 78.5 ± 8.2 at follow-up compared to 68.3 ± 9.6 in the control group (mean difference: 10.2, 95% CI: 7.6–12.8, *p* < 0.001). Within-group improvements were 20.2 ± 7.5 points for intervention versus 10.5 ± 6.8 points for control (between-group difference in change: 9.7, *p* < 0.001).

**Figure 4:**
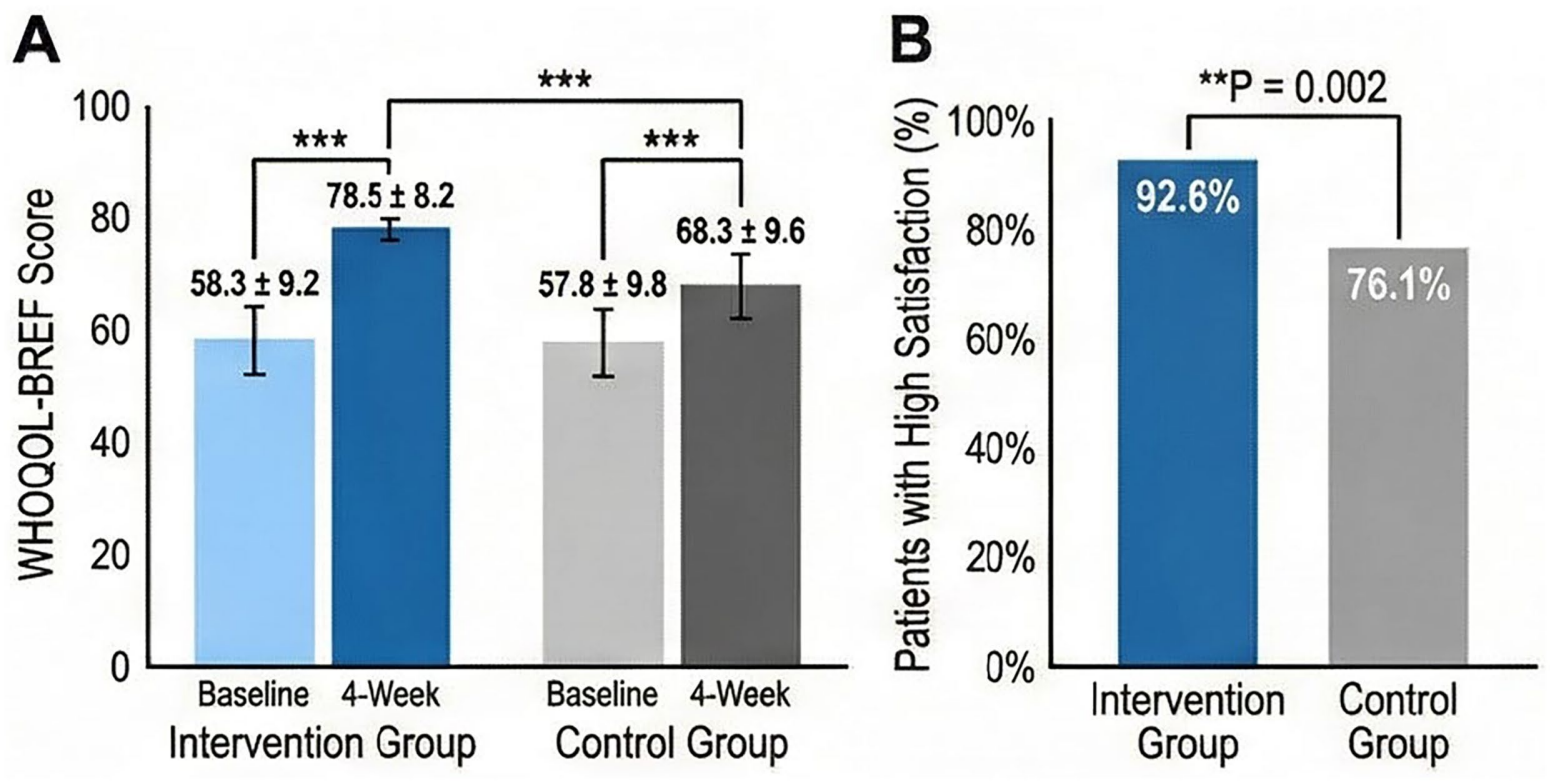
Comparison of quality of life (WHOQOL-BREF) and patient satisfaction at the 4-week follow-up between the intervention and control groups. ***p < 0.001. **(A)** WHOQOL-BREF Score; **(B)** Patients with High Satisfaction.

Patient satisfaction rates were significantly higher in the intervention group (92.6% reporting high satisfaction vs. 76.1% in control, *p* = 0.002). Specific satisfaction domains showing greatest differences included communication accessibility (95.7% vs. 72.8%, *p* < 0.001), symptom management support (91.5% vs. 68.5%, *p* < 0.001), and care coordination (93.6% vs. 70.7%, *p* < 0.001) (Figure 4).

**Figure 5:**
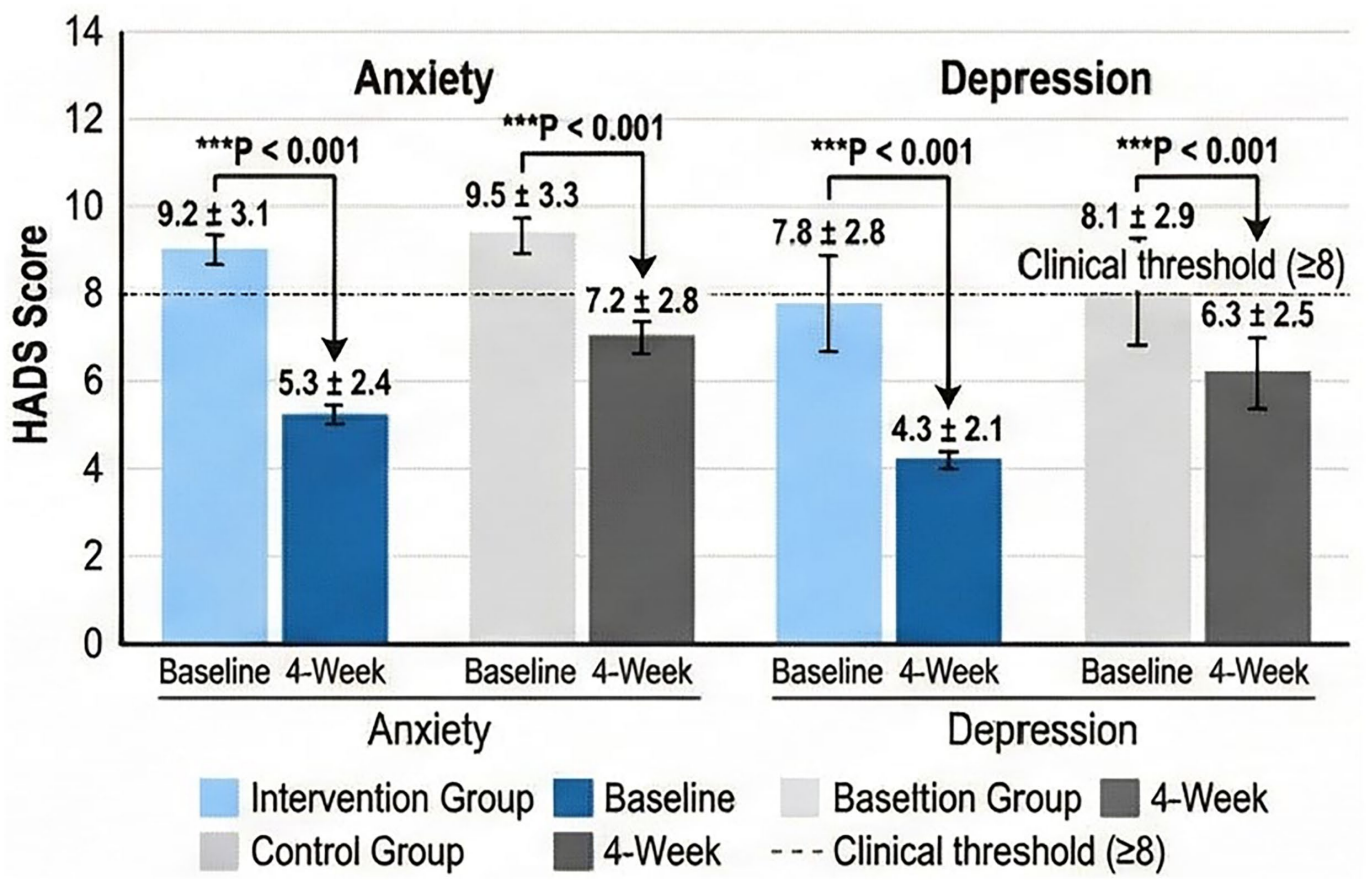
Changes in anxiety (HADS-A) and depression (HADS-D) scores from baseline to the 4-week follow-up in the two groups. ***p < 0.001.

Psychological outcomes demonstrated significant between-group differences. HADS-Anxiety scores decreased from 9.2 ± 3.1 to 5.3 ± 2.4 in the intervention group (change: −3.9 ± 2.2) compared to a decrease from 9.5 ± 3.3 to 7.2 ± 2.8 in controls (change: −2.3 ± 2.0), with significant between-group difference in change (*p* < 0.001). Similarly, HADS-Depression scores showed greater reduction in the intervention group (change: −3.5 ± 2.1 vs. −1.8 ± 1.9, *p* < 0.001). The proportion of patients with clinically significant anxiety (HADS-A ≥ 8) at follow-up was 18.1% in the intervention group versus 38.0% in controls (*p* = 0.003) (Figure 5).

Self-care ability measured by ESCA demonstrated significant improvement in both groups, with greater gains in the intervention group. The intervention group achieved mean ESCA scores of 138.5 ± 12.8 at follow-up compared to 118.2 ± 14.5 in controls (*p* < 0.001). The within-group improvement was 40.0 ± 10.5 points for intervention versus 21.0 ± 9.8 points for control (between-group difference in change: 19.0, *p* < 0.001). All four ESCA dimensions showed significantly greater improvement in the intervention group: self-concept (*p* < 0.001), self-care responsibility (*p* < 0.001), self-care knowledge (*p* < 0.001), and self-care skills (*p* < 0.001).

### 3.4 Multivariable analysis

**Figure 6:**
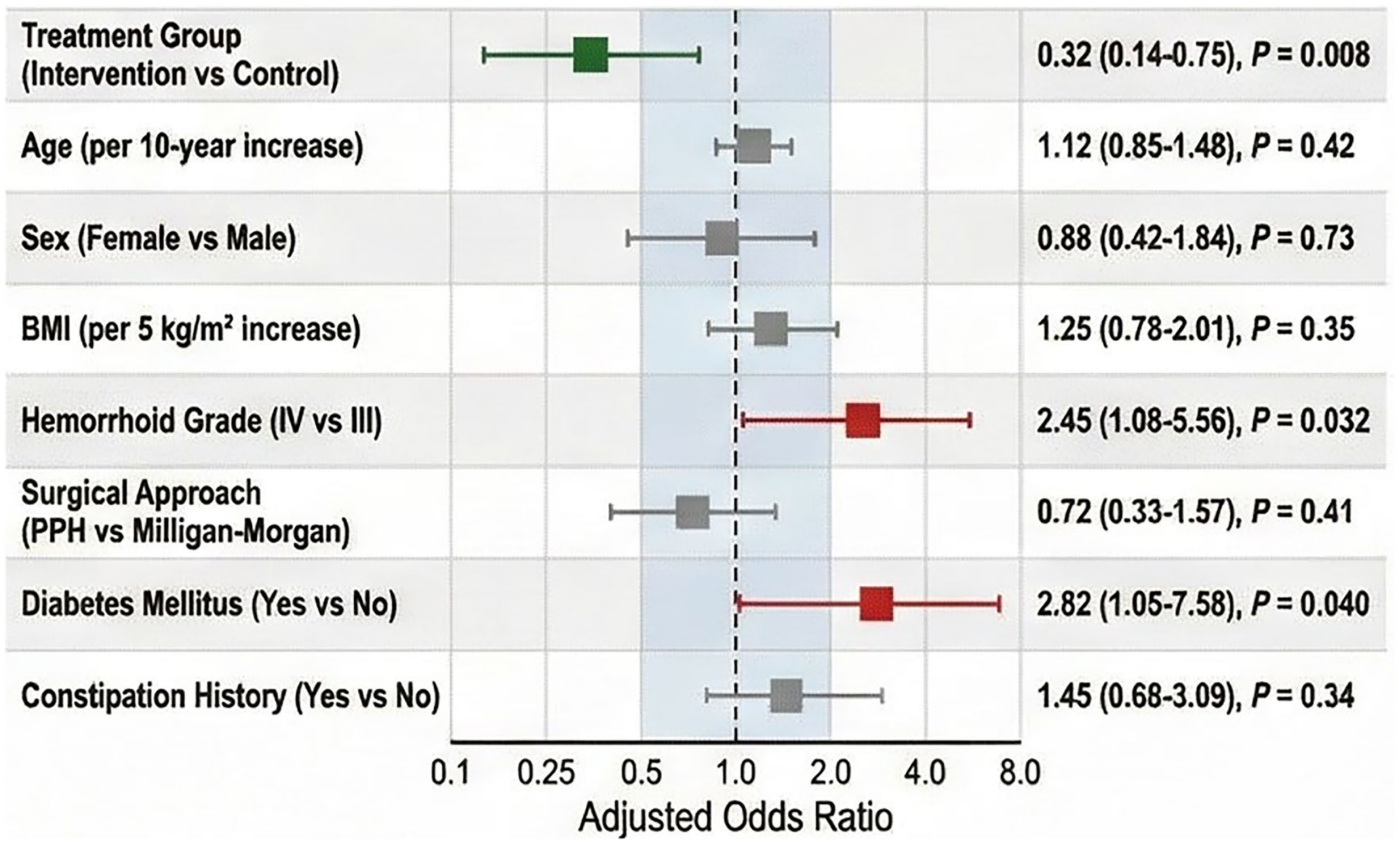
Forest plot of multivariable logistic regression analysis for independent predictors of postoperative complications. Adjusted odds ratios (OR) and 95% confidence intervals (CI) are shown.

Multivariable logistic regression identified intervention group assignment as an independent predictor of reduced overall complications (adjusted OR: 0.32, 95% CI: 0.14–0.75, *p* = 0.008) after controlling for age, sex, BMI, hemorrhoid grade, surgical approach, diabetes, and constipation history. Other significant predictors included grade IV hemorrhoids (adjusted OR: 2.45, 95% CI: 1.08–5.56, *p* = 0.032) and diabetes mellitus (adjusted OR: 2.82, 95% CI: 1.05–7.58, *p* = 0.040) (Figure 6).

## 4 Discussion

The present retrospective cohort study provides compelling evidence that an internet-based multidisciplinary management model delivering continuity of care significantly improves postoperative outcomes in patients undergoing surgery for mixed hemorrhoids. Our findings demonstrated that patients receiving the intervention achieved substantially shorter wound healing times, reduced complication rates, lower pain scores, enhanced quality of life, greater patient satisfaction, improved psychological wellbeing, and increased self-care capacity compared to those receiving conventional postoperative care. These results support the integration of digital health platforms with coordinated multidisciplinary approaches as an effective strategy for optimizing perioperative hemorrhoid management.

The observed 12.8% reduction in wound healing time (28.6 vs. 32.8 days) represents a clinically meaningful improvement with significant implications for patient recovery and healthcare resource utilization. This finding aligns with previous research demonstrating the positive impact of enhanced postoperative monitoring and education on surgical wound healing outcomes (17). Haravuori et al. (18) reported similar acceleration of wound healing in colorectal surgery patients receiving structured telemedicine follow-up, attributing improvements to earlier identification of healing impediments and timely intervention modifications. The continuous monitoring capability of our internet-based platform enabled prompt recognition of factors potentially delaying healing, including inadequate wound care technique, suboptimal dietary adherence, and excessive physical activity, allowing for immediate corrective guidance (19). Furthermore, the multidisciplinary nature of the intervention ensured that wound healing was addressed holistically, with dietitians optimizing nutritional status, rehabilitation therapists guiding appropriate activity progression, and nurses providing detailed wound care instruction (20).

The significant reduction in overall complication rates (1.1% vs. 7.6%) observed in the intervention group has important implications for patient safety and healthcare economics. The 63% relative risk reduction for complications exceeds benefits reported in studies examining single-component interventions such as telephone follow-up alone or standard educational materials (16). The comprehensive nature of our multidisciplinary intervention likely contributed to this substantial effect through multiple complementary mechanisms. Real-time symptom monitoring with automated alerts enabled early detection of developing complications before progression to clinical significance, facilitating preemptive intervention (21). The accessibility of healthcare providers through the digital platform reduced barriers to care-seeking, potentially preventing minor concerns from escalating to serious complications (22). Additionally, the psychological support component may have contributed to better overall recovery by addressing anxiety and stress responses that can impair immune function and wound healing processes (23).

The observed improvements in pain outcomes are particularly noteworthy given that postoperative pain represents a primary concern for hemorrhoid surgery patients and significantly influences recovery experience and satisfaction (24). While initial postoperative pain scores were comparable between groups, the intervention group demonstrated accelerated pain resolution, with differences becoming apparent by day 7 and persisting through day 14. This trajectory suggests that the intervention’s benefits on pain relate not to immediate analgesic effects but rather to optimized pain management strategies, enhanced self-care behaviors, and reduced anxiety-related pain amplification (25). The ability to communicate concerns about pain control and receive prompt adjustment recommendations through the digital platform likely contributed to more effective pain management compared to the limitations of scheduled clinic visits in the control group.

The substantial improvements in quality of life and patient satisfaction observed in our study underscore the importance of patient-centered outcomes in evaluating healthcare interventions (26). The 10.2-point difference in WHOQOL-BREF scores between groups exceeds established minimal clinically important differences for this instrument, indicating meaningful impact on patients’ daily functioning and wellbeing (27). High satisfaction rates (92.6%) in the intervention group, particularly regarding communication accessibility and care coordination, reflect the value patients place on continuous connectivity with healthcare providers during vulnerable recovery periods. These findings support theoretical frameworks emphasizing that patient engagement and empowerment through technology-enabled care delivery enhance perceived care quality and treatment outcomes (28).

The psychological benefits demonstrated in our study, including reduced anxiety and depression, deserve particular attention. Surgical procedures commonly trigger significant psychological distress, with hemorrhoid surgery patients potentially experiencing additional concerns related to the intimate nature of the condition and fears regarding functional outcomes (29). The 50% reduction in clinically significant anxiety prevalence in the intervention group suggests that the combination of accessible support, consistent information, and psychological counseling effectively addressed these concerns. The improvement in self-care ability represents an additional important finding, as enhanced self-efficacy and health management capacity have been associated with sustained positive health behaviors and outcomes beyond the immediate postoperative period (30).

The clinical implications of this study support the adoption of internet-based multidisciplinary management models as standard components of perioperative hemorrhoid care. Healthcare institutions should consider developing digital platforms enabling continuous patient-provider communication, structured symptom monitoring, and coordinated multidisciplinary engagement. The economic benefits of reduced complications, shorter healing times, and decreased emergency utilization may offset implementation costs while simultaneously improving patient experience. Training programs should prepare healthcare providers for effective virtual care delivery and multidisciplinary collaboration within digital environments.

Several limitations warrant consideration. The retrospective design introduces potential selection bias, as patients with greater technological literacy or engagement motivation may have been more likely to receive and comply with the intervention. The single-center setting limits generalizability to other healthcare contexts with different patient populations or resource availability. Regarding external validity, the current intervention depends on stable internet access, smartphone operation, and sufficient multidisciplinary telehealth support, which may restrict its transferability to rural or resource-limited regions with inadequate digital infrastructure or shortages of specialized staff; tailored simplified versions with offline backup or community-based assistance may be needed for wider implementation The 4-week follow-up period, while sufficient to assess short-term outcomes, cannot determine long-term effects on recurrence rates or sustained quality of life improvements. Patient-reported outcomes may be subject to recall bias and social desirability effects. The inability to blind participants or outcome assessors in this pragmatic intervention context may have influenced some subjective outcome measurements. Additionally, the 4-week follow-up duration is relatively short, which may be insufficient to capture important long-term outcomes after mixed hemorrhoid surgery, including hemorrhoid recurrence, delayed complications (such as late anal stenosis and chronic anal pain), and long-term quality of life. Therefore, the long-term efficacy and safety of this internet-based multidisciplinary continuity care model cannot be fully confirmed in the current study, and the findings should be interpreted cautiously when extrapolating to long-term prognosis. Second, although multiple primary and secondary outcomes were assessed, we used a hierarchical structure and avoided unadjusted pairwise comparisons. We acknowledge the potential risk of type I error from multiple comparisons but focused on clinically meaningful differences, and key results remained robust after multivariable adjustment. Information bias from patient-reported questionnaires was minimized using validated tools and fixed, researcher-supervised assessment time points.

In conclusion, this retrospective cohort study provides evidence that an internet-based multidisciplinary management model for continuity of care significantly improves postoperative outcomes in mixed hemorrhoid patients, including accelerated wound healing, reduced complications, decreased pain, enhanced quality of life, and improved psychological wellbeing. These findings support the integration of digital health technologies with coordinated multidisciplinary approaches in perioperative anorectal surgical care. Prospective randomized controlled trials with extended follow-up are warranted to confirm these findings and establish definitive clinical recommendations.

## Data Availability

The original contributions presented in the study are included in the article/supplementary material, further inquiries can be directed to the corresponding author.

## References

[1] Al-Masoudi RO Shosho R Alquhra D Alzahrani M Hemdi M Alshareef L . Prevalence of hemorrhoids and the associated risk factors among the general adult population in Makkah, Saudi Arabia. Cureus. (2024) 16:e52366. doi: 10.7759/cureus.51612, 38318578 PMC10840063

[2] Menconi C Marino F Bottini C la Greca G Gozzo C Losacco L . Evaluation and management of chronic anorectal and pelvic pain syndromes: Italian Society of Colorectal Surgery (SICCR) position statement. Tech Coloproctol. (2024) 28:69. doi: 10.1007/s10151-024-02943-1, 38907168

[3] van Tol RR Kleijnen J Watson AJM Jongen J Altomare DF Qvist N . European Society of Coloproctology: guideline for haemorrhoidal disease. Color Dis. (2020) 22:650–62. doi: 10.1111/codi.14975, 32067353

[4] Davis BR Lee-Kong SA Migaly J Feingold DL Steele SR. The American Society of Colon and Rectal Surgeons clinical practice guidelines for the management of hemorrhoids. Dis *Colon Rectum*. (2018) 61:284–92. doi: 10.1097/DCR.0000000000001030, 29420423

[5] Nakhla N Hospattankar A Siddiqui K Bridgeman MB. Improving hemorrhoid outcomes: a narrative review and best practices guide for pharmacists. Pharmacy (Basel). (2025) 13:105. doi: 10.3390/pharmacy13040105, 40863702 PMC12389048

[6] Yang Y Feng K Lei Y Qiu L Liu C Li G. Comparing the efficacy and safety of different analgesic strategies after open hemorrhoidectomy: a systematic review and network meta-analysis. Int J Color Dis. (2023) 38:4. doi: 10.1007/s00384-022-04294-5, 36609578

[7] Ljungholm L Edin-Liljegren A Ekstedt M Klinga C. What is needed for continuity of care and how can we achieve it?–perceptions among multiprofessionals on the chronic care trajectory. BMC Health Serv Res. (2022) 22:686. doi: 10.1186/s12913-022-08023-0, 35606787 PMC9125858

[8] Carfora L Foley CM Hagi-Diakou P Lesty PJ Sandstrom ML Ramsey I . Patients’ experiences and perspectives of patient-reported outcome measures in clinical care: a systematic review and qualitative meta-synthesis. PLoS One. (2022) 17:e0267030. doi: 10.1371/journal.pone.0267030, 35446885 PMC9022863

[9] Pereira Gray DJ Sidaway-Lee K White E Thorne A Evans PH. Continuity of care with doctors—a matter of life and death? A systematic review of continuity of care and mortality. BMJ Open. (2018) 8:e021161. doi: 10.1136/bmjopen-2017-021161, 29959146 PMC6042583

[10] Kruse CS Krowski N Rodriguez B Tran L Vela J Brooks M. Telehealth and patient satisfaction: a systematic review and narrative analysis. BMJ Open. (2017) 7:e016242. doi: 10.1136/bmjopen-2017-016242, 28775188 PMC5629741

[11] Schwamm LH Estrada J Erskine A Licurse A. Virtual care: new models of caring for our patients and workforce. Lancet Digit Health. (2020) 2:e282–5. doi: 10.1016/S2589-7500(20)30104-7, 32382724 PMC7202848

[12] Monaghesh E Hajizadeh A. The role of telehealth during COVID-19 outbreak: a systematic review based on current evidence. BMC Public Health. (2020) 20:1193. doi: 10.1186/s12889-020-09301-4, 32738884 PMC7395209

[13] Ljungqvist O Scott M Fearon KC. Enhanced recovery after surgery: a review. JAMA Surg. (2017) 152:292–8. doi: 10.1001/jamasurg.2016.4952, 28097305

[14] Wullaert L Voigt KR Verhoef C Husson O Grünhagen DJ. Oncological surgery follow-up and quality of life: meta-analysis. Br J Surg. (2023) 110:655–65. doi: 10.1093/bjs/znad022, 36781387 PMC10364539

[15] Jeong JH. Mobile application-based monitoring in post-breast reconstruction surgery: current trends and future directions. Arch Aesthetic Plast Surg. (2025) 31:1–9. doi: 10.14730/aaps.2025.01284

[16] Pooni A Brar MS Anpalagan T Schmocker S Rashid S Goldstein R . Home to stay: a randomized controlled trial evaluating the effect of a postdischarge mobile app to reduce 30-day readmission following elective colorectal surgery. Ann Surg. (2023) 277:e1056–62. doi: 10.1097/SLA.0000000000005527, 35815882

[17] Zhang M Zhu L Lin SY Herr K Chi CL Demir I . Using artificial intelligence to improve pain assessment and pain management: a scoping review. J Am Med Inform Assoc. (2023) 30:570–87. doi: 10.1093/jamia/ocac231, 36458955 PMC9933069

[18] Haravuori H Suomalainen L Marttunen M. Quality of life in adolescents and young adults after traumatic experience. Psychiatr Fenn. (2016) 47:32–49. doi: 10.1097/00004356-200509000-00007

[19] Zigmond AS Snaith RP. The hospital anxiety and depression scale. Acta Psychiatr Scand. (1983) 67:361–70. doi: 10.1111/j.1600-0447.1983.tb09716.x, 6880820

[20] Kearney BY Fleischer BJ. Development of an instrument to measure exercise of self-care agency. Res Nurs Health. (1979) 2:25–34. doi: 10.1002/nur.4770020105, 254279

[21] Leo DG Buckley BJ Chowdhury M Buckley BJR Harrison SL Isanejad M . Interactive remote patient monitoring devices for managing chronic health conditions: systematic review and meta-analysis. J Med Internet Res. (2022) 24:e35508. doi: 10.2196/35508, 36326818 PMC9673001

[22] Tan SY Sumner J Wang Y Wenjun Yip A. A systematic review of the impacts of remote patient monitoring (RPM) interventions on safety, adherence, quality-of-life and cost-related outcomes. NPJ Digit Med. (2024) 7:192. doi: 10.1038/s41746-024-01182-w, 39025937 PMC11258279

[23] Greer JA Pirl WF Park ER Lynch TJ Temel JS. Behavioral and psychological predictors of chemotherapy adherence in patients with advanced non-small cell lung cancer. J Psychosom Res. (2008) 65:549–52. doi: 10.1016/j.jpsychores.2008.03.005, 19027443 PMC4028043

[24] Ni K Zhu J Ma Z. Preoperative anxiety and postoperative adverse events: a narrative overview. Anesthesiol Perioper Sci. (2023) 1:23. doi: 10.1007/s44254-023-00019-1

[25] Mavros MN Athanasiou S Gkegkes ID Polyzos KA Peppas G Falagas ME. Do psychological variables affect early surgical recovery? PLoS One. (2011) 6:e20306. doi: 10.1371/journal.pone.0020306, 21633506 PMC3102096

[26] Doyle C Lennox L Bell D. A systematic review of evidence on the links between patient experience and clinical safety and effectiveness. BMJ Open. (2013) 3:e001570. doi: 10.1136/bmjopen-2012-001570, 23293244 PMC3549241

[27] Skevington SM Lotfy M O'Connell KA. The World Health Organization's WHOQOL-BREF quality of life assessment: psychometric properties and results of the international field trial. Qual Life Res. (2004) 13:299–310. doi: 10.1023/B:QURE.0000018486.91360.00, 15085902

[28] Hibbard JH Greene J. What the evidence shows about patient activation: better health outcomes and care experiences; fewer data on costs. Health Aff (Millwood). (2013) 32:207–14. doi: 10.1377/hlthaff.2012.1061, 23381511

[29] Flannery RB. Psychological trauma and the trauma surgeon. Psychiatry Q. (2022) 93:27–33. doi: 10.1007/s11126-020-09862-y, 33219925

[30] Yang Y Niu L. Effect of early rehabilitation nursing on motor function and living ability of patients with traumatic brain injury based on Orem’s self-care theory. Comput Intell Neurosci. (2022) 2022:1–9. doi: 10.1155/2022/7727085, 36120688 PMC9477576

