# Supplementary material for: Impact of internet-based multidisciplinary management model on continuity of care intervention for postoperative outcomes in patients with mixed hemorrhoids: a retrospective cohort study
Source: Front Med (Lausanne). 2026 Jun 5;13:1817812. doi: 10.3389/fmed.2026.1817812 (PMC13278973; doi:10.3389/fmed.2026.1817812)
Supplement: Supplementary file 1 [file Supplementary_File_1.docx]

| **CASE REPORTING FORM (CRF)**  **To be filled by doctor and sent to District Immunization Officer within 24 hours *Mandatory Field** | | | | |
| --- | --- | --- | --- | --- |
| ****Study Case ID:****  (Mixed Hemorrhoids Postoperative Management) | | | | |
| **Section A: Reporter and Study Details** | | | | |
| Name of doctor reporting / filling this form* | | | Contact phone number* | |
| E mail* | | | Place of present posting* | |
| Designation* | | | Address of present posting: | |
| Reporting Date (when this form is prepared)* | | | Patient’s surgery date (case enrolled / interviewed)* | |
| Notified by (Name)*: | | | Date notified (case enrolled)* | |
| Designation of notifier (please circle): Research Nurse / Surgeon / Study Coordinator / OthersSpecify: | | | | |
| Address of session/study site*: | | Village or Urban area: | | |
| Block Name: | | District: | | |
| State: | | Study Group*: ☐ Intervention Group ☐ Control Group | | |
| Intervention / Treatment Source: Hospital standard care / Study protocol / Others (specify): | | | | |
| Date of Intervention Start*: | | Time of Intervention Start: | | |
| Study Setting*: | | Inpatient / Outpatient / Follow-up / Others (specify): | | |
| Type of Intervention Site: | | Fixed clinic / home-based / mobile / others (specify) | | |
| **Section B: Patient details** | |  | | |
| Patient Name* | | Date of Birth* (DD/MM/YYYY) | | |
| Age | | Sex* ☐ Male ☐ Female | | |
| Mother’s Name | | Spouse/Father’s Name* | | |
| Complete Address* with landmarks (Street name, house number, village, block, Tehsil, PIN No, Telephone No. etc.) | | | | |
| PIN | | PHONE* | | |
| Clinical Characteristics | |  | | |
| 1. Hemorrhoid Grade (Goligher) | | ☐ III ☐ IV | | |
| 2. Surgical Procedure | | ☐ Milligan-Morgan ☐ PPH | | |
| 3. Comorbidities | | ☐ Hypertension ☐ Diabetes ☐ Constipation ☐ None | | |
| **Section C: Postoperative Outcomes & Follow-up** | | | | |
| Baseline VAS Pain Score | Pre-op / Day 0 | | | /100 |
| Postoperative VAS Pain Score | POD 1 | | | /100 |
| Postoperative VAS Pain Score | POD 3 | | | /100 |
| Postoperative VAS Pain Score | POD 7 | | | /100 |
| Postoperative VAS Pain Score | POD 14 | | | /100 |
| Wound Healing Time | Days post-op | | | days |
| Complications (Bleeding/Infection/Retention/Stenosis/Incontinence) | 4-week follow-up | | | ☐ Yes ☐ No (specify: ________) |
| WHOQOL-BREF Quality of Life Score | 4-week follow-up | | | /100 |
| HADS Anxiety Score | 4-week follow-up | | | /21 |
| HADS Depression Score | 4-week follow-up | | | /21 |
| ESCA Self-Care Ability Score | 4-week follow-up | | | /172 |
| Patient Satisfaction | 4-week follow-up | | | ☐ Satisfied ☐ Fair ☐ Dissatisfied |
